# Refinement of an Algorithm to Detect and Predict Freezing of Gait in Parkinson Disease Using Wearable Sensors

**DOI:** 10.3390/s25010124

**Published:** 2024-12-28

**Authors:** Allison M. Haussler, Lauren E. Tueth, David S. May, Gammon M. Earhart, Pietro Mazzoni

**Affiliations:** 1 Program in Physical Therapy, School of Medicine, Washington University in St. Louis, St. Louis, MO 63108, USA; 2 Department of Neurology, School of Medicine, Washington University in St. Louis, St. Louis, MO 63108, USA; 3 Department of Neuroscience, School of Medicine, Washington University in St. Louis, St. Louis, MO 63108, USA; 4 Department of Neurology, College of Medicine, The Ohio State University, Columbus, OH 43210, USA

**Keywords:** Parkinson disease, freezing of gait, wearable sensors, rehabilitation, gait assessment

## Abstract

Freezing of gait (FOG) is a debilitating symptom of Parkinson disease (PD). It is episodic and variable in nature, making assessment difficult. Wearable sensors used in conjunction with specialized algorithms, such as our group’s pFOG algorithm, provide objective data to better understand this phenomenon. While these methods are effective at detecting FOG retrospectively, more work is needed. The purpose of this paper is to explore how the existing pFOG algorithm can be refined to improve the detection and prediction of FOG. To accomplish this goal, previously collected data were utilized to assess the prediction ability of the current algorithm, the potency of each FOG assessment task(s) for eliciting FOG, and the maintenance of detection accuracy when modifying the sampling rate. Results illustrate that the algorithm was able to predict upcoming FOG episodes, but false positive rates were high. The Go Out and Turn-Dual Task was most potent for eliciting FOG, and the 360-Dual Task elicited the longest duration of FOG. The detection accuracy of the pFOG algorithm was maintained at a sampling rate of 60 Hz but significantly worse at 30 Hz. This work is an important step in refining the pFOG algorithm for improved clinical utility.

## 1. Introduction

Parkinson disease (PD) is the second most common neurodegenerative disease, affecting nearly one million individuals in the United States [1,2]. One of the most debilitating symptoms of PD is freezing of gait (FOG) [3], defined as a “brief episodic absence or marked reduction in the forward progression of the feet despite the intention to walk [4]”. FOG is estimated to impact up to 80% of individuals with PD over the course of the disease [5], but is still one of the most poorly understood symptoms. It is episodic in nature and highly variable, which makes assessment in the clinic difficult.

Whether a patient experiences FOG can be determined using the New Freezing of Gait Questionnaire (NFOG-Q), which is a self-report tool [6]. While this questionnaire is quick and easy to administer, it is subjective, relies on accurate recall, and does not correlate well with in-lab assessments of FOG [7]. Other methods include video review by a trained movement disorders expert, which is time-consuming, cumbersome, and focuses on retrospective identification of FOG episodes [8]. Accurate, real-time assessment of FOG is important because FOG is associated with decreased quality of life and reduced independence, leading to increased sedentary behavior, social withdrawal, and fear of falling [9,10]. The unpredictable nature of FOG episodes can lead individuals to experience falls, resulting in further reduced mobility and injury [11,12]. A better understanding of when and how people with PD experience FOG episodes would be useful for both clinicians and people with PD.

Objective measurement of FOG is a growing area of interest in PD research and developing additional methods to detect and evaluate FOG is a high research priority [13]. A leading approach is the use of wearable sensors. Various wearable sensor approaches have been used to objectively measure other common PD symptoms such as tremor [14,15] and dyskinesia [16]. For FOG, wearable sensors are often used in combination with custom algorithms to assess FOG in both lab and home settings [8,17]. Wearable sensors are useful for assessing FOG because they are portable and provide objective, quantitative data on how a person is moving before, during, and after an FOG episode. These sensors often include accelerometers and gyroscopes, which can be placed on the shank [18], foot [19], and/or trunk [20]. Methods to detect FOG from sensor data range from the use of thresholds [18] to machine learning techniques [21]. Much of the wearable sensor research in PD focuses on using these wearables to detect FOG episodes, with only 7 out of 74 articles in a recent review mentioning prediction [22]. While algorithms have been developed that can detect FOG episodes with reasonable accuracy [22], important next steps for this work include refining these methods to improve detection accuracy and extending these algorithms to yield prediction of FOG episodes.

Previous work in our group demonstrated the utility of wearable sensors in combination with the Probability of Freezing of Gait (pFOG) algorithm, developed by Prateek et al. [19], to assess FOG both in the clinic and at home [17]. While this methodology allowed for retrospective identification of FOG, we are also interested in whether the pFOG algorithm could be used to predict upcoming FOG episodes. Prediction of FOG episodes could be very useful from a clinical perspective. Knowledge of an impending FOG episode in real time could provide people with PD and clinicians with enough time to implement prevention or warning strategies. Additionally, we explored which tasks elicited FOG episodes most consistently across participants to improve the assessment of FOG. Finally, the pFOG algorithm was originally tested with sensors using a high sampling rate, which greatly impacts battery life and limits the types of sensors that can be utilized. Lowering the sampling rate could extend the battery life of these wearable sensors and would allow for reduced user burden and the ability to collect more data. The purpose of the present study is to refine our current pFOG algorithm and testing methods to improve the prediction and detection of FOG episodes in people with PD.

## 2. Materials and Methods

Data from a previously collected dataset were analyzed for the present study [17]. Basic inclusion criteria were as follows: (1) a diagnosis of “clinically definite PD”; (2) a history of FOG in the past two months according to the treating movement disorders neurologist; and (3) self-reported ability to walk 100 feet with or without an assistive device. Major exclusion criteria were as follows: (1) interfering medical conditions; and (2) a Mini-Mental Status Exam (MMSE) score < 24. Demographics are listed in Table 1. For full inclusion and exclusion criteria, see May et al. [17].

Participants completed one in-person visit at the Washington University in St. Louis Movement Science Research Center. During this visit, participants were first assessed with the Movement Disorders Society Unified Parkinson’s Disease Rating Scale (MDS-UPDRS-III) and then completed the NFOG-Q to ensure they met inclusion criteria. Next, participants engaged in a number of walking tasks and simulated instrumental activities of daily living (IADLs) while wearing three inertial measurement unit (IMU) sensors. One sensor was worn on the top of each foot, and one was worn on the left hip. The first six participants wore Physilog 6 sensors (Gait up SA, Lausanne, Switzerland) sampling at 128 Hz, and the remaining fourteen participants wore ActiGraph GT9X Link sensors (ActiGraph, Pensacola, Florida) sampling at 100 Hz. We changed sensors mid-study due to supply chain limitations during the COVID-19 pandemic. The walking tasks, hereafter referred to as clinic FOG tasks, and the simulated IADLs were designed to elicit FOG in-lab. The clinic FOG tasks included the following: (1) Hallway Pivot, (2) Go Outside and Turn (GOT), and (3) 360-Degree Turn (360). These were performed as single and dual-tasks. For the dual-task format, participants were asked to perform the clinic FOG task while simultaneously counting backwards by threes from a random 3-digit number. The simulated IADLs included the following: (1) vacuuming; (2) emptying a dishwasher; (3) sitting in a recliner with a footstool; and (4) navigating the hallways, doorways, and elevators of the clinic building to reach a destination, designed to simulate navigating a public building. Participants completed the sitting in a recliner task to allow us to validate that no FOG episodes were detected by the sensors when the participant was not walking. This was performed as a verification that the sensors were accurately differentiating walking vs. non-walking time. For further description of both sets of tasks, see May et al. [17].

### 2.1. FOG Prediction

For the present prediction analysis, IMU data from the in-lab clinic FOG and IADL tasks were assessed for all participants (ActiGraph and Physilog wearers) who experienced FOG episodes during the lab session. ActiGraph files were up-sampled to 128 Hz to allow all files to be processed through the pFOG algorithm at the same sampling rate. For this dataset, video review was considered the “gold standard” for detecting FOG during the in-lab portion and was performed by a licensed physical therapist with experience assessing people with PD. True positive FOG episodes were time points when both the algorithm and the video rater detected a freezing event. True negative FOG episodes were time points when neither the algorithm nor the video rater detected a freezing event. Only true positive FOG events were included in this analysis. After FOG events were identified, probability values for the three seconds leading up to a freeze were extracted using the statistical programming language R [23] and patterns were assessed. This three-second time frame was chosen because a few seconds may be enough time for delivery of a warning or intervention to prevent the impending FOG episode but is not too far removed from the episode. The ability of the algorithm to predict an upcoming FOG event was defined as an instance when the probability values, leading up to a freeze rose monotonically above a specific probability threshold at least one second before a true-positive freezing episode. Seven different thresholds were explored in this analysis, chosen around the center value of 0.35, which is half the threshold for a computer-defined FOG event. The thresholds explored included the following: 0.2, 0.25, 0.3, 0.35, 0.4, 0.45, and 0.5. The true prediction rate (fraction of algorithm-identified FOG events predicted) and false positive prediction rate were calculated with each threshold for each participant and then averaged across participants to determine the sensitivity and specificity of the pFOG algorithm’s ability to predict future FOG episodes. These rates were calculated using custom routines in Igor Pro. Different threshold values were explored to determine the best criteria for maximum FOG detection with the lowest false positive rate for this group of individuals with PD.

### 2.2. FOG Provocation by Task

Using the video review described above, we examined the effectiveness of each task in eliciting FOG. We assessed which task(s) elicited FOG in the most participants and which task(s) provoked the longest duration of FOG summed across participants.

### 2.3. Sampling Rate

For the present sampling rate analysis, raw IMU data from the in-lab clinic FOG and simulated IADL tasks for the 14 participants who wore ActiGraph GT9X Link sensors were down-sampled from 100 Hz to both 60 Hz and 30 Hz. Data were only analyzed for participants who wore the ActiGraph GT9X Link sensors to maintain consistency with the starting sampling rate. The down-sampled values of 60 Hz and 30 Hz were chosen because ActiGraph GT9X Link sensors have a sampling frequency range of 30 Hz to 100 Hz. Data were down-sampled with a 21-point sync filter with a Hanning window (“resample” operation in Igor Pro v. 8, Wavemetrics).

After down-sampling, each participant’s IMU data were re-processed using the pFOG algorithm to detect FOG events. For this analysis, the procedure, hardware, and software were identical to how the original 100 Hz IMU data were processed using the pFOG algorithm in May et al. [17]. The pFOG algorithm was implemented in Python (version 3.6) on a 2016 Macbook Pro laptop running MacOS version 10.13 and Darwin Kernel version 20. The process of calculating the probability that a freeze is occurring involves a series of computations that include detection and filtering modules [19]. FOG events were defined as any time either foot was above an FOG probability threshold of 0.7. FOG events separated by less than two seconds were merged into one FOG episode.

The accuracy of the algorithm to detect FOG episodes was calculated at each sampling rate using an existing Python programming language [24] script. As in previous work [17], accuracy was defined as the ratio of the amount of time defined as true positive FOG (defined above) plus the amount of time defined as true negative FOG divided by the total length of time during the in-lab session. Accuracy was calculated for the session as a whole, as well as for each clinic FOG task and simulated IADL individually. For the clinic FOG tasks, single-task and dual-task formats were divided out and treated as individual conditions. The accuracy percentages for the in-lab session as a whole and each simulated IADL and clinic FOG task for the ActiGraph GT9X Link participants were then averaged across participants. Normality of the data was established through visual assessment of histograms and QQ-plots, and calculation of skewness and kurtosis values. Overall accuracy at each sampling frequency was then compared using a one-way ANOVA with an alpha of 0.05 to determine potential differences. If significant effects were noted, post hoc Tukey tests were run to parse out differences. Additionally, the sensor-based percentage of active walking time spent freezing (Sensor-Based %WTSF) was calculated as in previous work by dividing the total amount of time spent freezing by the total amount of active time and multiplying by 100 [17]. The Sensor-Based %WTSF was calculated for the whole in-lab visit (clinic FOG tasks plus simulated IADLs) as well as separately for the clinic FOG tasks and simulated IADLs.

## 3. Results

### 3.1. FOG Prediction

**Figure 1:**
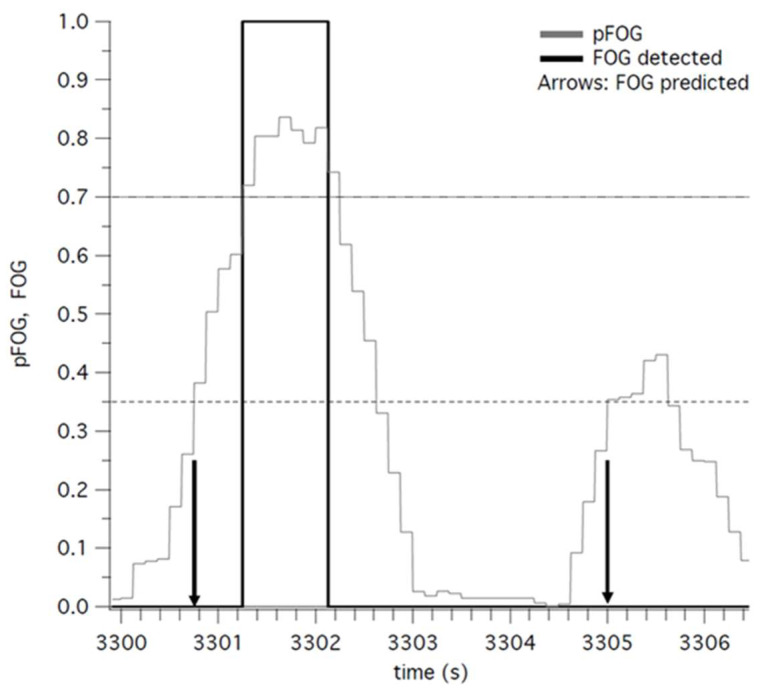
Examples of accurate and inaccurate predictions of FOG events for one participant. Prediction occurs when pFOG (gray trace) monotonically rises above 0.35 (arrows). At time 3300.8 s this prediction is followed, 0.5 s later, by the algorithm’s detection of an FOG event (pFOG rises above 0.7; black trace). At time 3305, the prediction is inaccurate because it is not followed by an FOG event detection.

Of the 19 participants in the existing dataset, 14 experienced FOG episodes during the in-lab clinic FOG and IADL tasks and are therefore included in the prediction analysis. An example of true prediction and false prediction is illustrated in Figure 1. The graph shows two instances (arrows) when pFOG rose monotonically above 0.35 and thus we predicted the upcoming detection of an FOG event. This prediction was correct at time 3300.8 s (left arrow), as it was followed by FOG detection around 0.5 s later. The prediction was incorrect at time 3305.0 s (right arrow), as it was not followed by the detection of an FOG event. The mean true prediction rate (fraction of algorithm-identified FOG events predicted) and false positive prediction rate at each probability threshold are presented in Table 2. Overall, the true prediction rate decreases as the threshold for prediction increases. The false positive prediction rate illustrates the same pattern, also decreasing as the threshold increases. For example, the threshold of 0.2 demonstrates an 80% true positive prediction rate, meaning that 80% of algorithm-detected freezes were predicted by monotonic rises above the threshold of 0.2. However, the false positive prediction rate for a threshold of 0.2 is 75%, meaning that 75% of monotonic rises above the threshold of 0.2 were false alarms.

### 3.2. FOG Provocation by Task

Each task’s ability to provoke FOG is presented in Table 3. The GOT-DT task elicited FOG events in the highest number of participants (n = 16 of 19). The 360-Degree Turn DT task elicited the longest duration of FOG summed across participants (78 s).

### 3.3. Sampling Rate

**Figure 2:**
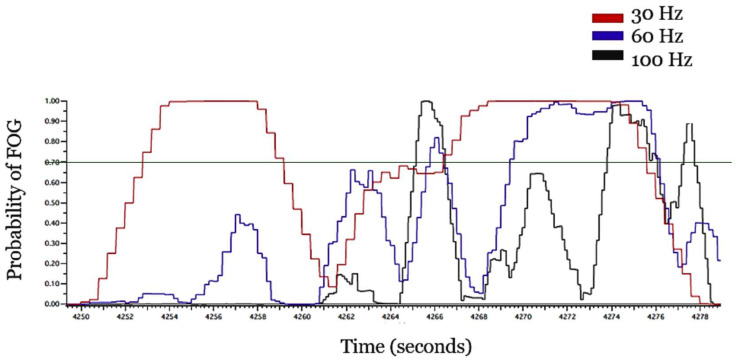
Snapshot of probability of FOG output at 100 Hz, 60 Hz, and 30 Hz. The various colored lines represent the algorithm probability value outputs for each sampling rate. The original 0.7 probability threshold to define an FOG event is marked in green.

Thirteen participants who wore the ActiGraph GT9X Link sensors fully completed the FOG assessment and are included in the present sampling rate analysis. The hip sensor was included in the initial study design for the purpose of filtering out sedentary time. Sedentary time was filtered out in the original analysis [17] and is therefore also filtered out here. Figure 2 illustrates a snapshot of the algorithm probability value outputs for each of the sampling rates tested, demonstrating that lowering the sampling rate alters this output. Visually, lowering the sampling rate from 100 Hz to 30 Hz reduces the nuance among the probability value outputs, essentially smoothing out important variability.

The mean accuracy percentages for all tasks combined across all participants (n = 13) for 100 Hz, 60 Hz, and 30 Hz were 89%, 87%, and 71%, respectively (Table 4). Accuracy percentages for the individual IADL tasks and the clinic FOG tasks (separated by single task and dual task format) are listed in Table 4. Data were normally distributed. A one-way ANOVA revealed that there was a statistically significant difference in overall accuracy between sampling rates (F2, 36 = 23.67, *p* < 0.001). Post Hoc Tukey HSD tests revealed significant accuracy differences between 30 Hz and 100 Hz (*p* < 0.001) and 60 Hz and 30 Hz (*p* < 0.001), but no significant accuracy differences between 100 Hz and 60 Hz (*p* = 0.68). Overall, accuracy percentages were higher for IADL tasks than for clinic FOG tasks. Sensor-based %WTSF is reported in Table 5. Participants in this sample generally spent more time freezing in the clinic FOG tasks.

## 4. Discussion

The results of these analyses demonstrate the pFOG algorithm can be improved and applied to new contexts. The results from the prediction analysis indicate the algorithm can detect an upcoming FOG episode using the chosen cut-offs for pFOG (Table 2). While the true prediction rates are above chance with every threshold explored, the false positive prediction rates are still very high. However, the purpose of predicting an upcoming FOG episode is to allow people with PD and clinicians time to implement prevention strategies such as an auditory or vibratory cue. Therefore, if the intervention strategy is minimally disruptive, such as a buzz on a watch, it may not be too bothersome to have false positive warnings if the true detection rate is high. The goal is to give people with PD warnings of an upcoming FOG episode so that they can prepare themselves and potentially prevent a fall. Given the prevalence of falls and injuries caused by FOG, any intervention strategy that reduces falls is worth exploring. One potential issue with the high false positive prediction rate is that individuals may start to ignore the warnings if they are coming too frequently. Further work in this area should explore personalized thresholds that use an individual’s own data to determine the best cutoff that maximizes FOG prediction while minimizing false positives. FOG varies from person to person and much work is needed to achieve proper FOG warnings that can be implementable in real-world applications. The next step is to assess individual gait and FOG detection patterns to improve personalized prediction.

When exploring the potency of the clinic FOG tasks to elicit FOG, we noted that the GOT and 360-DT elicited the greatest number of FOG events and the longest total duration of freezing, respectively. This is useful for clinicians to know, as FOG is often difficult to assess in the clinic due to its unpredictable nature. Turning is also a common self-reported trigger for FOG and can be somewhat effective in provoking FOG as measured by visual assessment [25]. While the sensors struggled to pick up FOG during turning, the video raters successfully identified these episodes. Future work should explore methods for improving sensor detection of FOG during turning, as this analysis demonstrates that turning is a potent provocation for FOG.

Additionally, while the clinic FOG and IADL tasks were designed to elicit FOG, participants did perform them in a controlled environment. Our past work suggests a strong correlation between sensor-based active walking time spent freezing during the in-person lab session and sensor-based active walking time spent freezing while wearing the sensors at home [17]. However, there was no video review comparison for home data. Future work could implement person-worn cameras that record foot motion at home to collect data for a “gold standard” real-world comparison to improve real-life generalizability. Video recording outside of the lab presents privacy concerns that would need to be addressed though. Furthermore, while this sample captured individuals across the disease span with varied FOG severity, this wearable sensor and algorithm method could be tested in larger samples with differing FOG triggers and comorbidities.

The lack of a significant difference in detection accuracy between 60 Hz and 100 Hz suggests that FOG detection is not hindered by lowering the sampling rate to 60 Hz. However, accuracy does decrease significantly when the sampling rate is lowered to 30 Hz. This knowledge is useful, as sensors that sample at lower frequencies are often less expensive and may have improved battery life. Saving battery life may also reduce user burden in terms of needing to recharge devices at home. This may allow for the implementation of this algorithm in a wider clinical context.

Overall, accuracy percentages were higher for the IADL tasks vs. clinic FOG tasks. This is promising as the IADL tasks are meant to represent the home environment and illustrate that wearable sensors may be a good way to assess FOG without a clinician present. Lower accuracy percentages for the clinic FOG tasks may be due to both the difficulty in defining an FOG event for a human rater as well as sensor complications with picking up steps during turning. For this analysis, the onset of an FOG event for video review is clear. However, the video review definition for the offset of an FOG event requires that the participant take two effective steps to resume walking. This definition is difficult to implement at times, particularly during the 360-Degree Turn task, as individuals with PD often take shuffling or sliding steps to turn around and may never reach the two effective steps criteria.

Many research groups are attempting to find ways to accurately detect and predict FOG. Several, like us, use IMUs [22], but others have used EEG [26,27,28]. While the EEG method is interesting in the lab setting, it would not be feasible in the community. Additionally, few groups have tested wearable sensor FOG detection in real-time [29,30,31]. This points to the importance of future work in this area, as real-time prediction and detection is the eventual goal of this line of research. Ultimately, our current work adds to this relatively small area of research, and we hope that together, this area of research can help spur innovation and improve the lives of people with PD and FOG. 

## 5. Conclusions

Overall, this work improves the utility of the pFOG algorithm. The prediction analysis demonstrates that this algorithm can predict upcoming FOG events. However, the high false positive rates should be addressed in future research. Tasks that involve turning are effective at provoking FOG and may be useful when measuring FOG severity in the clinic. Finally, lowering the sampling rate from 100 Hz to 60 Hz did not hinder sensor detection accuracy. The ability to lower the sampling rate without impacting accuracy suggests that potentially less expensive sensors with improved battery life may be implemented in combination with the pFOG algorithm, reducing user burden. The ultimate goal of FOG assessment and prediction of FOG is to better assess FOG and teach individuals about their freezing to help them implement strategies that reduce fall risk. Ultimately, reducing user burden and making this algorithm and wearable sensor method more cost-effective could allow for the collection of large datasets. These data could be collected without interfering with the individuals’ day-to-day lives and could greatly improve our understanding of FOG in PD.

## Figures and Tables

**Table 1:** Demographic information. (Full sample, *n* = 19). Mean (SD); [Range].

Participant Characteristics	
Age in Years	71.95 (7.21); [59–83]
MDS-UPDRS-III Score	41.89 (13.45); [20–73]
Levodopa Equivalent Daily Dose	955.2 (602.5); [150–2550]
New Freezing of Gait Questionnaire Score	18.63 (4.98); [0–26]
Sex (males, females)	M = 13, F = 6
Hoehn and Yahr Stages (Stage 2, 3, 4)	n = 8, n = 9, n = 2

**Table 2:** FOG prediction true positive and false positive prediction rates at each pFOG threshold. Sampling rate is 100 Hz. (Individuals who experienced true positive sensor FOG, n = 14). Mean (SD).

pFOG Threshold	True Prediction Rate	False Positive Prediction Rate
0.2	0.81 (0.13)	0.75 (0.12)
0.25	0.77 (0.16)	0.73 (0.13)
0.3	0.75 (0.16)	0.71 (0.14)
0.35	0.74 (0.15)	0.70 (0.14)
0.4	0.72 (0.15)	0.68 (0.14)
0.45	0.69 (0.16)	0.65 (0.15)
0.5	0.64 (0.18)	0.63 (0.15)

**Table 3:** Potency of clinic FOG tasks to elicit freezing. (Full sample, n = 19).

Task	Number of Participants Who Experienced FOG Event in Video Review (% of Sample)	Total Duration FOG Summed Across Participants (seconds)
360-Degree Turn	11 (58%)	58
360-Degree Turn DT	13 (68%)	78
GOT	14 (74%)	73
GOT-DT	16 (84%)	60
Hallway	12 (63%)	57
Hallway-DT	14 (74%)	50

**Table 4:** Accuracy at each sampling frequency for IADL tasks, clinic FOG tasks, and overall lab session. (ActiGraph GT9X wearers, n = 13). Mean (SD).

Task	100 Hz Accuracy	60 Hz Accuracy	30 Hz Accuracy
Sitting	98% (7%)	99% (4%)	100% (1%)
Vacuuming	95% (6%)	91% (7%)	83% (7%)
Dish Washer	94% (8%)	91% (7%)	71% (11%)
Community	96% (4%)	90% (7%)	66% (18%)
GOT	82% (17%)	75% (14%)	38% (18%)
GOT DT	81% (17%)	74% (16%)	38% (16%)
360-Degree Turn	73% (24%)	66% (21%)	38% (20%)
360-Degree Turn DT	63% (32%)	63% (26%)	43% (21%)
Hallway	87% (12%)	82% (10%)	58% (17%)
Hallway DT	85% (12%)	80% (10%)	46% (19%)
Overall Accuracy	89% (9%)	87% (7%)	71% (6%)

**Table 5:** Percent of sensor-based walking time spent freezing (%WTSF) for IADL tasks, clinic FOG tasks, and overall session. (ActiGraph GT9X wearers, n = 13). Mean (SD).

	100 Hz	60 Hz	30 Hz
%WTSF IADL	2% (4%)	9% (14%)	19% (20%)
%WTSF Clinic FOG	14% (17%)	23% (21%)	41% (25%)
Overall %WTSF	7% (8%)	14% (10%)	27% (12%)

## Data Availability

Deidentified data that support the findings in this publication may be available from the corresponding author (GE) upon reasonable request, subject to IRB restrictions and approval.
